# Physical Properties and Non-Isothermal Crystallisation Kinetics of Primary Mechanically Recycled Poly(l-lactic acid) and Poly(3-hydroxybutyrate-*co*-3-hydroxyvalerate)

**DOI:** 10.3390/polym13193396

**Published:** 2021-10-02

**Authors:** Luboš Běhálek, Jan Novák, Pavel Brdlík, Martin Borůvka, Jiří Habr, Petr Lenfeld

**Affiliations:** Department of Engineering Technology, Faculty of Mechanical Engineering, Technical University of Liberec, Studentská 1402/2, 461 17 Liberec, Czech Republic; jan.novak@tul.cz (J.N.); pavel.brdlik@tul.cz (P.B.); martin.boruvka@tul.cz (M.B.); jiri.habr@tul.cz (J.H.); petr.lenfeld@tul.cz (P.L.)

**Keywords:** poly (l-lactic acid), poly (3-hydroxybutyrate-*co*-3-hydroxyvalerate), mechanical recycling, non-isothermal crystallisation kinetics

## Abstract

The physical properties and non-isothermal melt- and cold-crystallisation kinetics of poly (l-lactic acid) (PLLA) and poly(3-hydroxybutyrate-*co*-3-hydroxyvalerate) (PHBV) biobased polymers reprocessed by mechanical milling of moulded specimens and followed injection moulding with up to seven recycling cycles are investigated. Non-isothermal crystallisation kinetics are evaluated by the half-time of crystallisation and a procedure based on the mathematical treatment of DSC cumulative crystallisation curves at their inflection point (Kratochvil-Kelnar method). Thermomechanical recycling of PLLA raised structural changes that resulted in an increase in melt flow properties by up to six times, a decrease in the thermal stability by up to 80 °C, a reduction in the melt half-time crystallisation by up to about 40%, an increase in the melt crystallisation start temperature, and an increase in the maximum melt crystallisation rate (up to 2.7 times). Furthermore, reprocessing after the first recycling cycle caused the elimination of cold crystallisation when cooling at a slow rate. These structural changes also lowered the cold crystallisation temperature without impacting the maximum cold crystallisation rate. The structural changes of reprocessed PHBV had no significant effect on the non-isothermal crystallisation kinetics of this material. Additionally, the thermomechanical behaviour of reprocessed PHBV indicates that the technological waste of this biopolymer is suitable for recycling as a reusable additive to the virgin polymer matrix. In the case of reprocessed PLLA, on the other hand, a significant decrease in tensile and flexural strength (by 22% and 46%, respectively) was detected, which reflected changes within the biobased polymer structure. Apart from the elastic modulus, all the other thermomechanical properties of PLLA dropped down with an increasing level of recycling.

## 1. Introduction

In modern civilisation, no one could imagine a day without the use of plastic goods. In 2019, more than 368 million tonnes of plastic were produced globally [1]. Over 90% of raw plastic is produced from fossil fuels [2] (non-renewable source). Most plastic products are non-biodegradable, are used only once, and are collected in landfills or energetically recycled [1]. It could cause substantial environmental problems associated with the growing population and area of use of these materials.

Consequently, the growing interest in scientific and industrial research is focused on developing materials with greater environmental sustainability—bioplastic. Advantageous mechanical properties and moderate thermal stability poly (lactic acid) PLA [3,4,5,6,7] and poly(3-hydroxybutyrate-*co*-3-hydroxyvalerate) (PHBV) [8,9,10,11] are amongst the most attractive biobased polymers for future applications. Unfortunately, high production costs are still one of the considerable limitations to the broader application of these materials [12]. However, the use of appropriate feedstock and processing technology offers potential for further improvement [13]. Although PLA and PHBV biobased polymers are made from renewable sources, the short end life cycle could not be guaranteed [14,15]. The different biodegradation rates and levels of bioplastics could be achieved during divergent biodegradation conditions [16,17,18]. Compared to PHBV, PLA is more sensitive to hydrolytic than microbial/enzymatic degradation [16,19]. Consequently, the hydrolytic degradation of PLA in a normal home composting process takes place to a limited extent. Therefore, it is advisable to explore the possibility of extending their service life before finally discarding them to biodisposal facilities. Recycling is a very effective method and could also contribute to reducing the final price [20].

Currently, two of the most widely used recycling methods of biobased polymers are chemical and mechanical recycling [8,21,22,23]. Chemical recycling consists of the depolymerisation of biobased polymers into constituent parts that could be used for further direct repolymerisation or as a feedstock for other applications. In mechanical recycling, which is the most profitable and widespread recycling method [24], the materials are grinded, crushed, or milled and eventually pelletised. The disadvantage of this method is the possibility of thermomechanical degradation [4,25]. This decomposition is initiated mainly by chain scissions and inter/intramolecular transesterifications, affecting the molar mass distribution and, subsequently, mechanical, thermal, and rheological properties, as well as discolorations [24,26]. Pillin et al. [27] investigated the influence of seven reprocessing cycles (injection moulding) on the mechanical, rheological, thermal, and structural properties of PLA. The published result revealed a significant decrease in molecular weight, glass transition temperature, and stress and strain at the brake and a significant increase in viscosity and crystallinity. Contrary, the tensile modulus was stable without any noticeable impact of reprocessing. Additionally, Badia et al. [25] reported the effect of thermomechanical degradation of PLA induced by the injection moulding process (five cycles). The chain scission caused a remarkable reduction in molecular weight that initiated morphology changes, as well as changes in viscosity, thermal properties, and dynamical–mechanic properties. Dia et al. [28] explained detected changes of molecular weight and mechanical properties with a generation of acidic molecules that accelerate the degradation process of PLA. Other aspects of chain scission could be a small value of the activation energy for thermal degradation (21–23 kJ/mol) and a high tendency of PLA to hydrolysis [29]. The study of Zaverl et al. [30] stated recycling potential of PHBV. After five reprocessing cycles, only a small decrease in mechanical and thermal properties was detected. The molecular weight of the polymer did not decrease drastically. Contrary to Shojaeiarani et al. [20], a significant decrease in molecular weight that caused a reduction in mechanical and thermal properties was observed. Many aspects could cause differences in thermomechanical degradation initiated by reprocessing, such as the concentration of reused material in the virgin matrix [24], the composition of additives in a biobased polymer [3,24,31,32], production process (shear stress), and lifetime history (oxidation, UV, etc.). From the material point of view, microstructure and morphology are fundamental aspects. Shojaeiarani et al. [20] investigated that chain scission occurs especially in long chains due to high shear rate and high temperature. Consequently, more significant changes in molecular weight that caused a higher decrease in mechanical and thermal properties of PLA and PHBV were detected in polymers with longer macromolecules. The conclusion is that the biobased polymers with lower molecular weight are more suitable for recycling than those with a higher molecular weight [24].

PLA is generally obtained by ring-opening polymerisation of lactide acid that could have two optical isomer forms: l-lactic and d-lactic. The stereoregular conformation and ratio of l- and d-lactic acid in PLA influence chain mobility and, subsequently, crystalline phase of PLA. Badia et al. [24] reported that low content (<0.5%) of d-lactic might show a fully amorphous morphology after several reprocessing steps. Contrary, the higher content of d-lactide (8%) promoted the creation of crystalline regions (38% and 53% after the second and seventh reprocessing phases, respectively). Chain scission creates shorter chains acting as nucleation centres. Thus, increasing the crystallisation kinetics rate could considerably affect the final properties of reused PLA [24,25].

Consequently, the current work was dedicated to evaluating the influence of specific injection moulding conditions on the crystallisation kinetics of reprocessed PLLA and PHBV. Our work used a relatively new approach to evaluate the non-isothermal kinetics of melt and cold crystallisations, which has been introduced by Kratochvíl and Kelnar [33]. This method eliminates the negative methodological factors associated with the use of conventional crystallisation models. Furthermore, the influence of reprocessing steps on the structural, mechanical, and thermal properties were evaluated.

## 2. Materials and Methods

The commercial poly(l-lactic acid) (PLLA) under the trade name of Luminy L130 was supplied by Total Corbion (Gorinchem, Netherlands). It is a medium flow homopolymer with stereochemical purity: a minimum of 99% L-898isomer, weight average molecular weight 170,000 g/mol, dispersity 1.65, glass transition temperature between 55 and 60 °C and melting temperature 175 °C. The commercial poly(3-hydroxybutyrate-*co*-3-hydroxyvalerate) (PHBV) under the trade name of NaturePlast PHI 002 by NaturePlast (Pantin, France), with weight average molecular weight 274,800 g/mol, dispersity 2.53, glass transition temperature of 5 °C, and melting temperature of 170 °C, was used. 

### 2.1. Sample Preparation 

The virgin and recycled materials were processed by injection moulding on an Arburg Allrounder 320 C hydraulic injection moulding machine (Arburg, Loßburg, Germany). Mechanical recycling was carried out on a knife mill Wanner C17.26sv (Wanner Technik, Wertheim, Germany). For all the injection moulding cycles, the parameters were kept constant. The temperature profile for PLLA was 170 °C, 175 °C, 180 °C, 185 °C, and 190 °C for the nozzle and for PHBV was 140 °C, 160 °C, 180 °C, 180 °C, and 185 °C for the nozzle. The injection speed was kept constant at 25 cm^3^/s for PLLA and 15 cm^3^/s for PHBV; the mould temperature was fixed at 20 °C for PLLA and 60 °C for PHBV; and a constant holding pressure of 45 MPa was applied to both biobased polymers. Biobased polymers were injected in a mould of type A normalised specimens according to ISO 3167. Before injection moulding, the virgin and recycled materials were dried in a Binder VD53 vacuum dryer (Binder, Tuttlingen, Germany). The residual moisture content was always less than 0.025%.

### 2.2. Differential Scanning Calorimetry (DSC)

Thermal properties and crystallisation kinetics were studied using a differential scanning calorimeter DSC 1/700 (Mettler Toledo, Greifensee, Switzerland), which was calibrated with indium and zinc standards. Experiments were carried on samples with different recycling cycles (1 to 7) under a constant nitrogen flow of 50 mL/min. Approximately 5 mg of sample specimens were prepared from the cross-section of the injection moulding parts on a rotating microtome Leica RM2255 (Leica Biosystem, Nußloch, Germany) were placed in 40 μL aluminium pans, sealed, and then placed in the DSC chamber. An empty pan was used as a reference. The specimens were heated from 0 to 200 °C, maintained at 200 °C for 3 min, cooled to 0 °C, and finally reheated to 200 °C at a heating rate of 10 °C/min. Melting peak temperature (*T_p,m_*), cold crystallisation peak temperature (*T_p,cc_*), premelt crystallisation peak temperature (*T_p,pc_*), melting enthalpy (Δ*H_m_*), cold crystallisation enthalpy (Δ*H_cc_*), and premelt crystallisation enthalpy (Δ*H_pc_*) obtained from the first and second heating scans and melt crystallisation peak temperature (*T_p,c_*) as well melt crystallisation enthalpy (Δ*H_c_*) determined from cooling scans were determined using the STARe software by Mettler Toledo (Greifensee, Switzerland). The samples were characterised at least in duplicate, and the averages were taken as representative values.

The crystallinity degree (*X_c_*) of the samples as a function of the recycling cycle was determined according to Equation (1), where $\Delta H_{m}^{0}$ is the theoretical melting enthalpy of the polymer assumed be 100% crystalline, $\Delta H_{m}^{0}$ = 106 J/g for PLLA [34], and $\Delta H_{m}^{0}$ = 146 J/g for PHBV [35].

(1) $$X_{c}\left(\%\right)=\frac{\Delta H_{m}-\Delta H_{pc}-\Delta H_{cc}}{\Delta H_{m}^{0}}\Delta 100$$

In a study of non-isothermal crystallisation kinetics (at a constant cooling rate of 10 °C/min), the relative crystallinity (*X_T_*) was determined according to Equation (2), where Δ*H_T_* is the heat of melt crystallisation, and *T*, *T*_0_, *T_∞_*, and Δ*H_c_* represent the temperature at any given moment, initial temperature, final temperature, and heat of melt crystallisation, respectively. The melt crystallisation time (*t*) is given by Equation (3), where *υ* is the constant cooling rate.

(2) $$X_{T}\left(\%\right)=\frac{\Delta H_{T}}{\Delta H_{c}}=\frac{\int_{T_{0}}^{T}\left(\frac{dH_{c}}{dT}\right)dT}{\int_{T_{0}}^{T_{\infty }}\left(\frac{dH_{c}}{dT}\right)dT}\Delta 100$$

(3) $$t\left(min\right)=\frac{T_{0}-T}{v}$$

**Figure 1:**
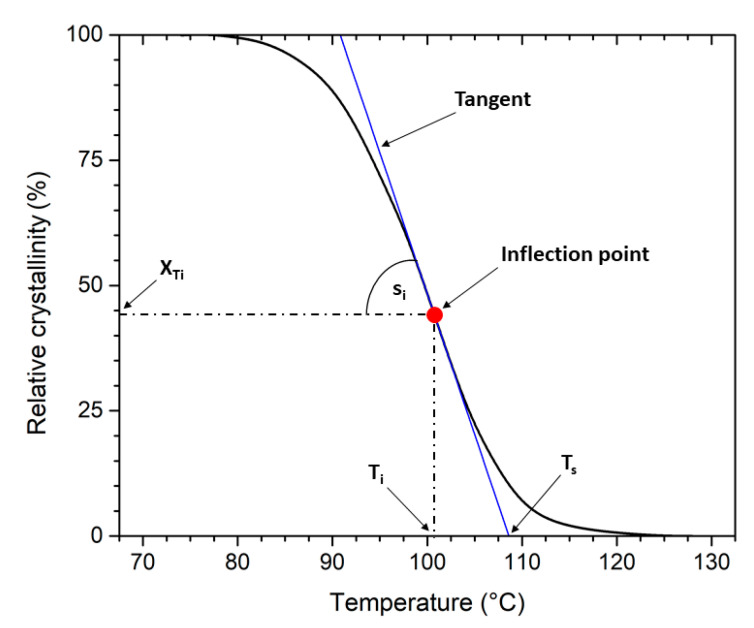
Schematic drawing of the non-isothermal crystallisation kinetics method by Kratochvíl and Kelnar [33]: cumulative curve.

The kinetics of non-isothermal crystallisation was also evaluated according to the method introduced by Kratochvíl and Kelnar [33]. The principle of the method is schematically shown in Figure 1. It is a new simple method for evaluation of non-isothermal crystallisation kinetics at a constant cooling rate. The procedure based on mathematical treatment of the DSC cumulative crystallisation curves (i.e., the dependence of relative crystallinity on temperature) at their inflection point provides four basic parameters: temperature of the start of crystallisation (*T_s_*), the temperature of maximum crystallisation rate (*T_i_*), the numerical value of the maximum crystallisation rate (*s_i_*) and final crystallinity after cooling from the melt (*X_c_*). This approach is particularly convenient for the comparison of the non-isothermal crystallisation kinetics of samples in series with one reference, a virgin polymer. The method provides the temperature of crystallisation start (*T_s_*) and maximum crystallisation rate (*T_i_*) with standard deviation 0.3 and 0.4 °C, respectively. Maximum crystallisation rate (s_i_) and final crystallinity (*X_c_*) have coefficients of variation 5.8 and 1.5%, respectively [33]. The repeatability of *T_s_*, *T_i_*, and *s_i_* improves with decreasing cooling rate. The method does not refer to any crystallisation models that are used in the evaluation of non-isothermal crystallisation (e.g., Ozawa, Nakamura, or Jeziorny extended Avrami kinetic model and others) [36,37] and eliminates the problem of exact setting of the starting time of crystallisation. As stated by Kratochvíl and Kelnar in their study [33], the parameters obtained sensitively describe the crystallisation process at the maximum rate, i.e., at relative crystallinities of about 35–45%. Thus, this method models real conditions encountered during processing polymeric materials well. The only prerequisite of successful application of the proposed method is a smooth cumulative crystallisation curve, i.e., well-developed crystallisation exotherm obtained by the DSC analysis.

### 2.3. Thermogravimetric Analysis (TGA)

Thermal experiments were performed using TGA2 instrument (Mettler Toledo, Greifensee, Switzerland). The specimens from each recycling cycle were prepared from the cross-section (approximately 5 mg) and heated from 50 to 600 °C with 10 °C/min heating rate under nitrogen atmosphere (flow: 50 mL/min). The decomposition temperature was determined at 5% weight loss (*T*_5_) and maximum weight loss (*T_max_*) determined from the derivate thermogravimetry (DTG) curve, which corresponds to the inflection point of the TGA curve. Specimens were subjected to five repetitive tests, and the averages were taken as representative values. 

### 2.4. Rheological-Flow Properties

Rheological-flow properties were determined by measuring the melt volume flow rate (MVR) at temperature 190 °C and under a load of 2.16 kg using a melt flow tester Ceast (Ceast, Torino, Italy), according to ISO 1133. Before measurement, the material was dried in a vacuum oven Binder VD53 (Binder, Tuttlingen, Germany) to a residual moisture content of less than 0.01%. Residual moisture was controlled on an HX204 halogen analyser (Mettler Toledo, Greifensee, Switzerland) at 130 °C.

### 2.5. Mechanical Properties

Tensile testing, flexural testing, and impact testing were carried on multipurpose test specimens of type A out at standard conditions 23/50, according to ISO 291. Specimens of type A are tensile test specimens, from which specimens with dimensions (80 × 10 × 4) mm have been obtained by machining to determine flexural and impact properties. Tensile modulus, tensile strength, and tensile strain at break values were determined according to ISO 527 standard using LabTest universal electromechanical testing instruments (Labortech, Opava, Czech Republic) with extensometer MFL-300B (Mess- & Feinwerktechnik, Velbert, Germany) with accuracy class ISO 9513, at a crosshead speed of 1 mm/min for determined tensile modulus and 5 mm/min for determined tensile strength and elongation at break, a 10 kN load cell, and gauche length of 50 mm. Flexural modulus and flexural strength values were determined according to ISO 178 (three-point loading test) using a Tinius Olsen H10KT testing machine (Tinius Olsen, Salfords, UK) at a crosshead speed of 2 mm/min. Charpy impact strength values were carried out following ISO 179-1/1eU on the equipment Resil 5.5 (Ceast, Torino, Italy), with a force of 5 J for PLLA and 2 J for PHBV. Mechanical analyses were repeated at least ten-fold, and the averages were taken as representative values. 

### 2.6. Heat Resistance Testing

Heat resistance testing of the recycles biobased polymers was performed with a ZwickRoell’s HDT/Vicat 6-300 Allround instrument (ZwickRoell, Ulm, Germany) by determining the Vicat softening temperature (VST) according to ISO 306. The dimensions of the specimens were (10 × 10 × 4) mm, VST were measured under a load of 50 N, and the temperature of the oil bath was raised by 50 °C/h. The load was applied to the specimens after 5 minutes of immersion in an oil bath at an initial temperature of 25 °C. Specimens were subjected to five repetitive tests. 

## 3. Results and Discussion

### 3.1. Thermal Properties and Structure of Recycled Biobased Polymers

**Figure 2:**
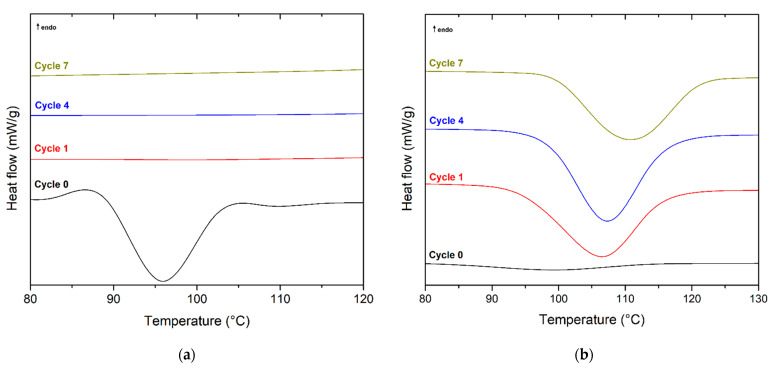
PLLA cold and melt crystallisation as a function of recycling cycle: (**a**) cold crystallisation (second heating); (**b**) melt crystallisation.

Unlike PHBV, PLLA is a biobased polymer with a characteristic slow melt crystallisation rate. During its subsequent heating, cold crystallisation, and sometimes premelt crystallisation, can be observed. These exothermal reactions have direct impact on the heat quantum necessary for crystal melting. As a result, it is necessary to subtract the values of cold crystallisation (Δ*H_cc_*) and premelt crystallisation (Δ*H_pc_*) from the final melting enthalpy (Δ*H_m_*) when calculating the degree of crystallinity of PLLA (*X_c_*) by DSC (Equation (1)). The resulting values of these endothermic and exothermic phase transformations, including the degree of crystallinity, are listed in Table 1 for PLLA and Table 2 for PHBV. The degree of crystallinity is evaluated from the first and second heating phases. The first heating phase reflects the supramolecular structure of the moulded part, which results from the cooling conditions during the moulding process, and the second heating phase reflects the crystallisation of the biobased polymer from the melt at a slow (laboratory) cooling rate of 10 °C/min after removing its thermal history. As a result of repeated mechanical recycling, a decrease in the values of premelt crystallisation enthalpy (Δ*H_pc_*) and cold crystallisation enthalpy (Δ*H_cc_*) was detected in PLLA. The slow cooling rate in the calorimeter resulted in a complete diminishing of the cold crystallisation during the first recycling cycle (Figure 2a). This effect is initiated by the significant increase in Δ*H_c_* values during the crystallisation of PLLA from the melt (Figure 2b). Shojaeiarani et al. [20], Pillin et al. [27], and Zaverl et al. [30] found that, for PLA and PHBV with a thermomechanical history of a successive recycling process, the weight average molecular weight, the number average of molecular weight, and the molecular weight distribution values decreased significantly.

Chain cleavage induced by thermomechanical degradation during repeated milling and PLLA injection moulding resulted in shorter polymer chains, which acted as nucleation centres. This premise potentially explains the increase in the melt crystallisation enthalpy (Δ*H_c_*) and final degree of crystallinity (*X_c_*). These results are partially inconsistent with the results reported in the study by Shojaeiarani et al. [20] and Badia et al. [25]. The reason is probably the high optical purity of PLLA, as both studies analysed poly (lactic acid), which contained a minimum of 4% mol of D-lactic enantiomer. The degree of crystallinity increased, on average, by three times in the seventh recycling cycle when PLLA melt was cooled at a rate of 10 °C/min. On the other hand, the increase in the degree of crystallinity observed in moulded parts reached only 48%. This difference is probably induced by various thermodynamic cooling conditions during solidification of the melt for moulded part and sample studied under laboratory conditions and is related to the limited movement of the macromolecules.

**Figure 3:**
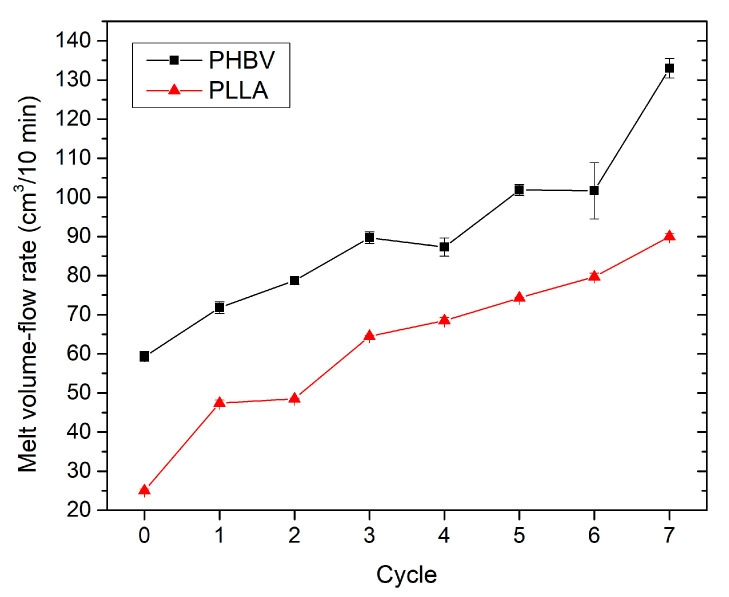
PLLA and PHBV melt volume flow rate as a function of recycling cycle.

Chain cleavage is evident from the rheological behaviour of the material (Figure 3). As the number of recycling cycles increases, the values of melt flow volume index (*MVR*) increase. This phenomenon is induced by the decrease in the molecular weight of the analysed biobased polymer. In the seventh PLLA recycling cycle, the melt flow volume index increased six-fold compared to the virgin material (pellets) and 3.6-fold compared to the moulded part made of the virgin material. For PHBV, an increase in *MVR* value was recorded, almost three-fold that of the virgin material in the form of pellets and 2.2-fold that of the moulded part made of the virgin material. Repeated mechanical recycling of biobased polymers causes a significant reduction in viscosity, especially for PLLA, which is an apparent symptom of a lower molecular weight induced by mechanical and thermal degradation. The negative effect of shear stress during the injection moulding process on the degradation of PLLA and PHBV is already evident from the change in the *MVR* values measured on the supplied commercial pellets and the crushed material milled from the moulded parts produced from the same pellets. This finding is consistent with the results of a study published by Pantani et al. [38], which deals with the degradation of PLA caused by different shear rates during its processing (extrusion and injection moulding). Between the pellets and the crushed material milled from the moulded parts produced from the same pellets, an increase in the *MVR* was observed by approximately 68% for PLLA and by 29% for PHBV. Abe [26] stated that a significant rise in melt volume flow rate for PHBV polymer could be attributed to the unzipping reaction in the biobased polymer structure through cis-elimination mechanism (Mclafferty arrangement) initiated just above its melting temperature. Similarly, the degradation of PLA is induced by the generation of acidic molecules acting as a catalyst to accelerate the degradation. According to Dai et al. [28], the considerable reduction in the molecular weight of PLA was instead associated with the thermal degradation caused by polymer chain scissions into linear and cyclic oligomers.

From the results of PLLA DSC analysis, is evident that the glass transition temperature (*T_g_* ~60 °C) did not decrease with increasing level of recycling (repeated thermomechanical loading). This finding is also confirmed by Badia et al. [25] and Żenkiewicz et al. [39]. On the other hand, Pillin et al. [27] found that, during the second recycling cycle, *T_g_* of the analysed PLA decreased. According to the Foxe–Flory relationship [40], this indicates that the decrease in molecular weight induced by reprocessing of PLLA was not significant enough to reduce the *T_g_* dramatically. 

The decrease in molecular weight affects melt crystallisation and, in addition to increasing the melt crystallisation enthalpy (Δ*H_c_*), the melt crystallisation temperature (*T_p,c_*) also increases, which means that the PLLA melt crystallises faster after repeated mechanical recycling (Figure 2b). A slight increase in Δ*H_c_* and *T_p,c_* was also observed for highly crystalline PHBV. In the case of the Δ*H_c_* value, an increase of only 2.5% was recorded for the seventh level of PHBV recycling, while for PLLA, this increase was almost tenfold (see Table 1 and Table 2). The biggest leap in crystallisation enthalpy was observed in the first recycling cycle. In PHBV, chain cleavage did not affect the phase transformation process in the frame of melt crystallisation, as in the case of PLLA. The resulting degree of crystallinity (*X_c_*) thus does not change for repeatedly processed PHBV (Table 2). A similar finding was made by Shojaeiarani [20]. On the contrary, this result contradicts the findings of Zaverl et al. [30], who recorded a decrease in the value of *X_c_* induced by repeated processing of PHBV (decrease by 20% in the fifth recycling cycle). The proposal can be introduced that this divergence can be caused by the different valerate content in the PHBV copolymer. Shojaeiarani et al. [20] found that chain cleavage occurs mainly in long chain biobased polymers. The higher rate of degradation of PLLA induced by thermomechanical loading during recycling is therefore probably caused by easier cleavage of the chains in the amorphous part of the supramolecular structure, which predominates in PLLA after injection into cold mould (20 °C).

**Figure 4:**
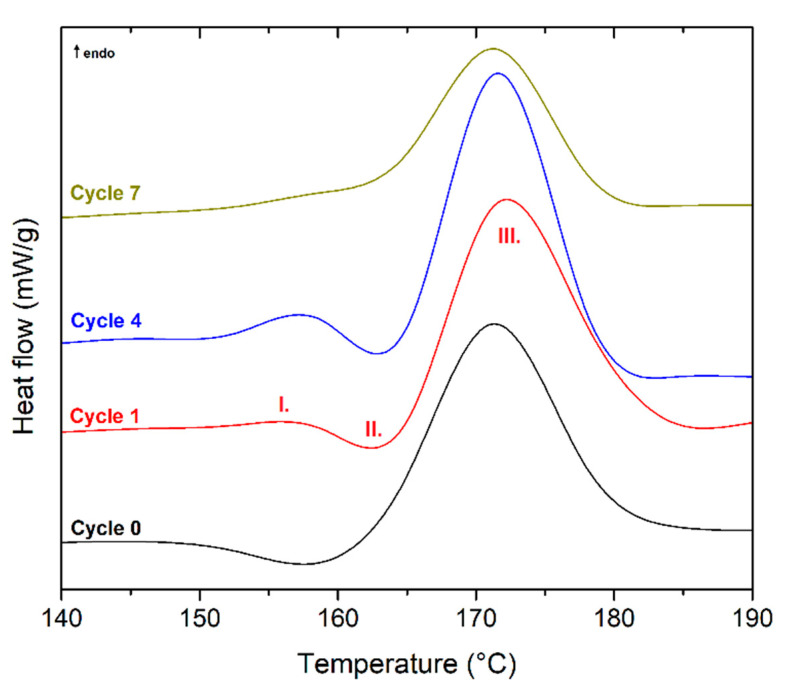
DSC curves (second heating) of PLLA as a function of recycling cycle.

From the results of PLLA DSC analysis (see Figure 4), it is evident that the endothermic phase transformation of melting (Peak III) is preceded by a sharper exotherm (Peak II) and a small and narrow endotherm (Peak I) in addition to cold crystallisation. The initial endotherm (Peak I) may be due to the melting of PLLA crystals with low thermal stability, i.e., paracrystalline or microcrystalline structures, formed at *T_p,c_* and/or in the temperature range of cold crystallisation. Successive structural recrystallisation and reorganisation (premelt crystallisation) may result in the appearance of an exotherm (Peak II) and a final endotherm (Peak III) [41,42]. A second alternative explanation for the presence of the endothermic overshoot in PLLA relies on the physical ageing of the rigid amorphous fraction, that is, the glassy fraction at the interface between the mobile amorphous fraction (*T_g_*) and the crystalline fraction [43]. Di Lorenzo [44] states that the position and dimension of the various peaks strongly depend on crystallisation temperature. 

Furthermore, the results showed that repeated mechanical recycling induced a gradual increase in melt crystallisation temperature (*T_p,c_*) (see Figure 2b). As the temperature *T_p,c_* increased, Peak I and Peak II moved to higher temperatures, Peak I became more pronounced, and Peak II was less intense (see Figure 4). In the seventh recycling cycle, Peak I coincided with Peak III. When crystallisation is performed at higher temperatures, due to repeated mechanical recycling, a single melting peak is always present, and its position is strongly affected by *T_p,c_*.

**Figure 5:**
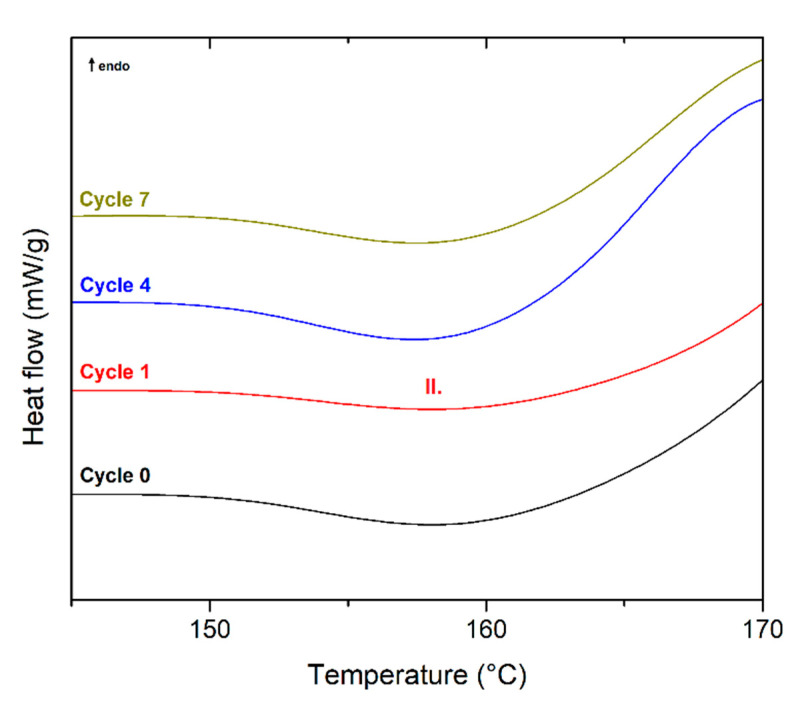
DSC curves (first heating) of PLLA as a function of recycling cycle.

The first DSC temperature cycle, which reflects the macromolecular structure of the moulded parts, shows that the endothermic melting transformation, in addition to cold crystallisation, is preceded by only exotherm (peak II) associated with structural recrystallisation caused by fast cooling rate of the melt after injection of PLLA into the cold mould (Figure 5).

Changes in the macromolecular structure of PLLA and PHBV induced by their gradual recycling were also evaluated by thermogravimetric analysis (TGA). In Table 3, the temperatures corresponding to 5% weight loss and maximum rate of weight loss for each biobased polymers recycling cycle are represented by *T_5_* and *T_max_*. Degradation of the moulded part made of virgin material (PLLA) began at 343 °C, and maximum weight loss occurred at 370 °C. In the case of PLLA, after seven recycling cycles, the material started to degrade by 71 °C earlier, i.e., at 272 °C, and the maximum weight loss occurred at 346 °C, i.e., 24 °C earlier than in the case neat injection moulded samples. PHBV also accelerated the onset of thermal degradation with increased recycling. However, the temperature changes were significantly lower than for PLLA. In the seventh recycling cycle, the temperature of T_5_ decreased by 6 °C and the *T_max_* by 5 °C, compared to the moulded part made of virgin material (PLLA). A similar decrease in degradation temperature, but significantly lower, was observed for PLA by Shojaeiarani et al. [20] and Żenkiewicz et al. [39] and for PHBV by Shojaeiarani et al. [20] and Zaverl et al. [30]. From the results, it can be stated that the higher molecular weight of PHBV increases its thermal stability during repeated processing. Carrasco et al. [45] and Crompton [46] also report that there is a linear relationship between thermal stability and the average molecular weight, in which the higher molecular weight can result in more thermally stable polymer materials.

### 3.2. Effect of Recycling on Non-Isothermal Crystallisation Kinetics 

**Figure 6:**
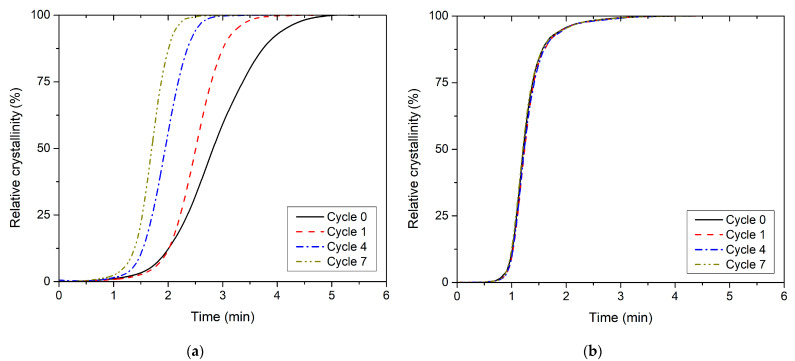
Relative crystallinity versus time for non-isothermal melt crystallisation (**a**) PLLA and (**b**) PHBV at cooling rate 10 °C/min.

The kinetics of non-isothermal crystallisation of PLLA and PHBV affected by their gradual recycling (in the range of seven recycling cycles) was monitored by DSC at a constant cooling rate of 10 °C/min. The trends in the behaviour of relative crystallinity (X_T_) over time was recorded (Figure 6). These graphical dependences were constructed from DSC measurements based on Equations (2) and (3). Table 4 shows the values of the half-time crystallisation (*t*_0.5_), defined as the time when crystallisation reached 50%. From the results, is obvious that the values of *t*_0.5_ for PLLA decrease gradually due to recycling; however, for PHBV, they remain unchanged. A decrease in *t*_0.5_ by 11% was recorded in the first PLLA recycling cycle and by 40% in the seventh recycling cycle. The decrease in *t*_0.5_ values caused by the gradual recycling of PLLA shows a trend that is typical in the case of faster cooling from the melt, as reported, for example, by Tarani et al. [47]. This effect is induced by the increase in the melt crystallisation temperature (*T_p,c_*) [48], which increases with gradual recycling for PLLA, while for PHBV, the change is insignificant (see Table 1 and Table 2). The reduction in *t*_0.5_ caused by repeated mechanical recycling confirms the cleavage of the chains, which act as nucleation centres during the non-isothermal crystallisation of PLLA.

**Figure 7:**
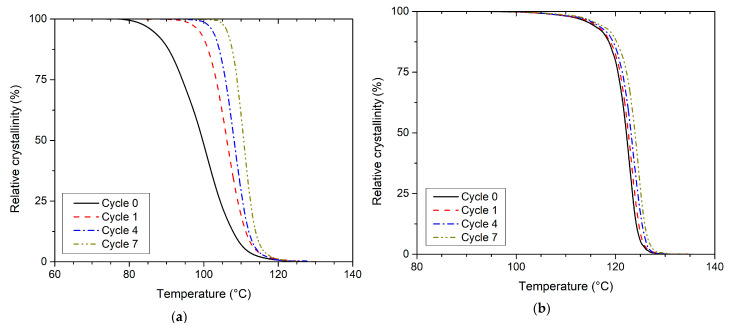
Relative crystallinity versus temperature for non-isothermal crystallisation (**a**) PLLA and (**b**) PHBV—cumulative crystallisation curves.

Table 4 lists several valuable parameters describing the non-isothermal kinetics of PLLA and PHBV crystallisation during their gradual recycling by the method introduced by Kratochvíl and Kelnar [33]. The starting point is the non-isothermal cumulative crystallisation curve (Figure 7), i.e., the dependence of relative crystallinity (*X_T_*) on temperature (*T*). The method is based on the fact that the slope of the tangent to the cumulative crystallisation curve at any point is directly proportional to the crystallisation rate at the corresponding temperature. The inflection point of the cumulative crystallisation curve (*s_i_*) is the point of maximum crystallisation rate. It is specified by temperature *T_i_* and relative crystallinity *X_Ti_* (Figure 1). The tangent slope of the cumulative crystallisation curve at the inflection point, in 1/°C, is the numerical value of the maximum crystallisation rate. The cross-section of the tangent with the T-axis determines the starting temperature of crystallisation (*T_s_*) [33]. From the above-mentioned parameters, the fact arises that the gradual recycling of PLLA increases the temperature of the melt crystallisation process onset and crystallisation rate. For the moulded part made of virgin PLLA, the maximum crystallisation rate (*s_i_*) of 5.6 1/°C was measured at 100.8 °C (*T_i_*), i.e., about 8 °C after the onset of crystallisation. At the same time, the PLLA after the seventh recycling cycle showed a maximum crystallisation rate 2.7 times higher (15.3 1 /°C), which already occurs at a temperature of 110.9 °C, i.e., approximately 3 °C after the onset of crystallisation. In the case of PHBV, the temperatures of *T_s_* and *T_i_* increase by gradual recycling, but the maximum crystallisation rate (*s_i_*) appears to be independent of the number of recycling cycles. In addition, the shift in temperatures *T_s_* and *T_i_* is significantly lower than in PLLA. The parameters obtained describing the crystallisation process at its maximum rate correspond to relative crystallinity of approximately 44–54% for PLLA and 37–43% for PHBV, which corresponds to the results of Kratochvíl and Kelnar [33]. Paukszta and Borysiak also observed an increase in the temperature and rate of melt crystallisation with non-isothermal cooling during repeated processing of the PP/PA6 polymer blend [49].

**Figure 8:**
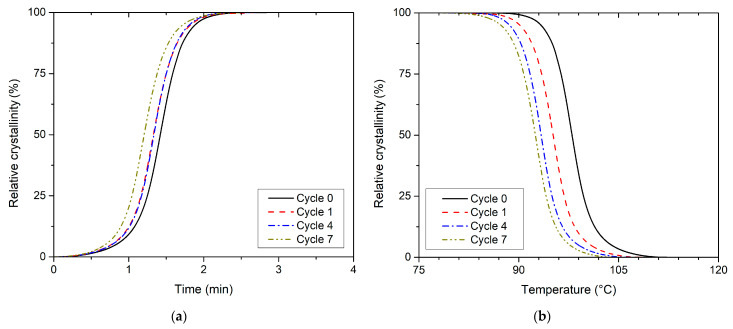
Relative crystallinity versus (**a**) time and (**b**) temperature for non-isothermal cold crystallisation PLLA.

Cold crystallisation occurred in the first phase of PLLA heating at a constant rate of 10 °C/min. The first heating reflects the thermal history of the PLLA created during the injection moulding process. The thermal characteristics are given in Table 5 and are based on DSC curves, on the graphical dependence of relative crystallinity on time, and the graphical dependences of relative crystallinity on temperature during cold crystallisation, respectively (Figure 8). 

Gradual recycling showed that the cold crystallisation peak temperature (*T_p,cc_*) and the temperature of cold crystallisation start (*T_s,cc_*), which was determined by non-isothermal heating according to the method introduced by Kratochvíl and Kelnar [33], shifted to lower temperatures. The difference between *T_p,cc_* and *T_s,cc_* between the recycling cycle 0 and 7 is approximately 5 °C. The evaluation of the non-isothermal kinetics of cold crystallisation further shows that repeated recycling does not affect the maximum rate of cold crystallisation, in contrast to melt crystallisation. Similar to the beginning of cold crystallisation (*T_s,cc_*), the temperature of the maximum rate of cold crystallisation (*T_i,cc_*) shifts to lower values. Additionally, the difference in *T_i,cc_* values between recycling cycle 0 and 7 is approximately 5 °C. The results thus showed that the heating of gradually recycled PLLA, in which the volume of crystalline structure formed during solidification from the melt did not meet the potential of this material (caused by rapid cooling of the material within injection mould), activates conformational processes of macromolecules at lower temperatures. These processes are associated with the cold crystallisation of PLLA. However, the rate of cold crystallisation and the enthalpy of cold crystallisation are consistent for an entire range of reprocessed PLLA that were analysed (see Table 1 and Table 5). The nucleation centres created by chain cleavage during repeated recycling of PLLA were thus fully utilised in melt crystallisation. The obtained parameters describing the PLLA cold crystallisation process at its maximum rate correspond to a relative crystallinity of approximately 47–51%.

### 3.3. Effect of Recycling on Mechanical Properties

**Figure 9:**
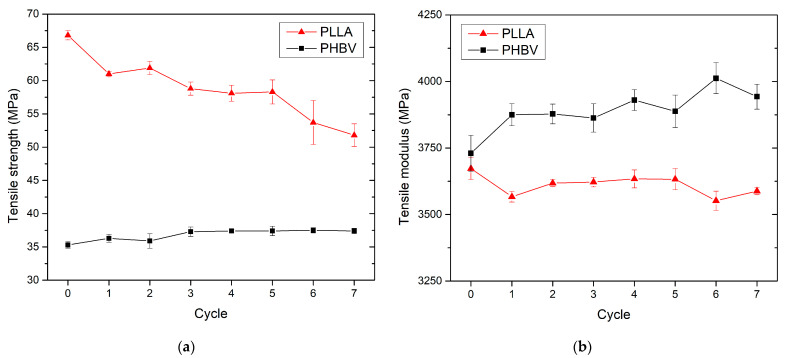
PLLA and PHBV tensile properties as a function of recycled cycle: (**a**) tensile strength; (**b**) tensile modulus.

**Figure 10:**
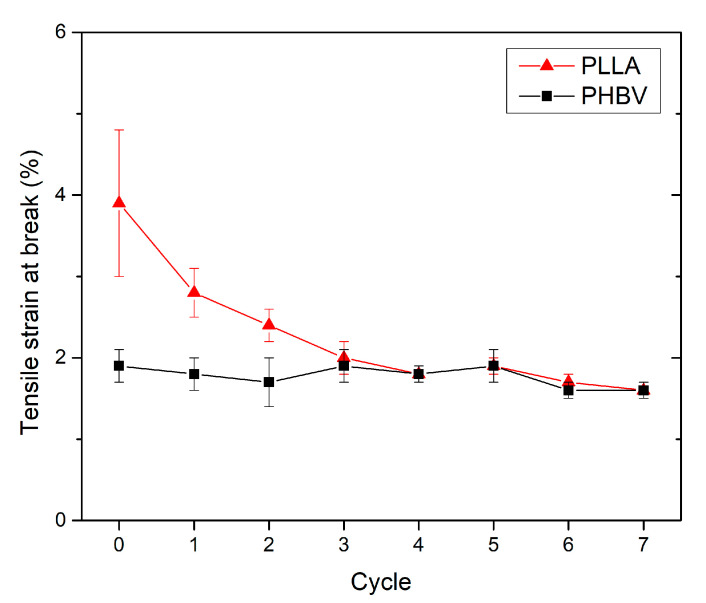
PLLA and PHBV tensile strain at break as a function of recycled cycle.

**Figure 11:**
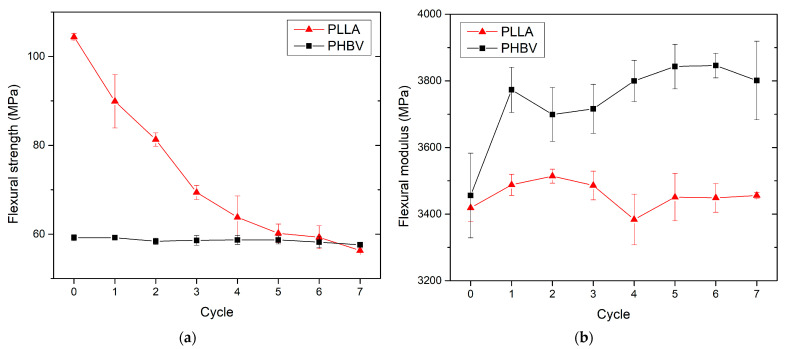
PLLA and PHBV flexural properties as a function of recycled cycle: (**a**) flexural strength; (**b**) flexural modulus.

**Figure 12:**
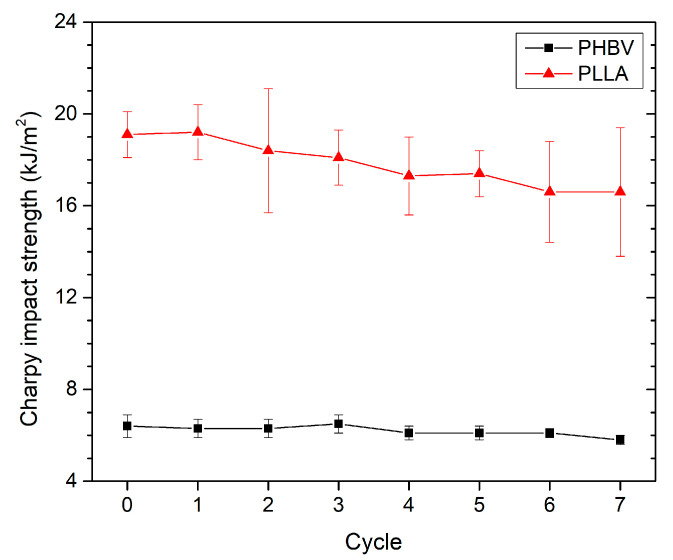
PLLA and PHBV Charpy impact strength as a function of recycled cycle.

The mechanical properties of virgin and recycled PLLA and PHBV biobased polymers were studied through tensile, flexural, and impact tests, and the results are summarised in Figure 9, Figure 10, Figure 11 and Figure 12. PLA exhibited significantly lower tensile and flexural strength with the increasing number of recycling cycles. In the seventh recycling cycle, an average decrease in tensile strength by 22% was recorded (Figure 9a) and flexural strength even by 46% (Figure 11a). The highest range of changes was recorded in the first recycling cycle, when a decrease in tensile strength reached almost 9%, and flexural strength dropped by 14%. The greatest decrease in tensile strength, comparing the first recycling cycle and the virgin material, was also recorded in PLA by Żenkiewicz et al. [39]. Changes in tensile strength and flexural strength of PHBV are statistically insignificant. In the seventh recycling cycle, an average decrease in flexural strength by almost 3% was recorded for PHBV, and quite contrary, a slight increase by almost 6% was recorded for tensile strength. A very similar trend, but not with such significant differences, was also observed by Shojaeiarani et al. [20]. A decrease in mechanical strength can probably be attributed to the lower molecular weight in the recycled polymers due to the deterioration of the polymer chains during successive injection moulding cycles. In general, the mechanical properties of biobased polymers are strongly dependent on molecular weight. Therefore, any changes in the polymer chain as a result of degradation induced by high temperature and shear within the injection moulding machine directly impact the mechanical properties. Tensile and flexural strength were significantly lower for reprocessed PLA as compared to their corresponding counterpart made of virgin material (as opposed to reprocessed PHBV), which is probably caused by the trend of decreasing molecular weight in recycled polymers indirectly observed by the change in viscosity and melt volume flow index, respectively (Figure 3).

The tensile and flexural modulus does not show significant changes in recycled PLLA (Figure 9b and Figure 11b). Considering the scattering of the measured values, these changes appear to be insignificant. These results were also confirmed by the study focused on the tensile modulus published by Pillin et al. [27] for recycled PLA containing 92% of L-lactide and 8% of D-lactide. Conversely, for recycled PHBV, an increase in the mean value of the modulus of elasticity in tension and bending was recorded (by about 6% and 10% in the seventh recycling cycle). The recycling process includes polymer crushing, melting, and its repeated shear stress, which leads to polymer chain degradation. However, thermal degradation in the melting process did not seem to contribute to a significant failure of the intermolecular bonds between the polymer chains, because the stiffness of the polymers did not show a decreasing trend with the increased number of recycling cycles.

The tensile test results showed that PLLA and PHBV exhibited a typical brittle fracture with strain values of less than 10% (around 2% to 4%). This effect occurs as a result of testing PLLA below the glass transition temperature. For PHBV, the brittle fracture is caused by the relatively high degree of crystallinity of the moulded part (about 60%, see Table 2). The brittleness of poly(hydroxybutyrate) (PHB) with the crystallinity degree puts into context a study by El-Taweel et al. [50]. El-Taweel et al. found that, above 40% crystallinity, PHB exhibited brittle fracture. Because the degree of crystallinity of recycled PHBV did not decrease, the tensile strain at break is almost constant in the frame of this study (Figure 10). Conversely, for recycled PLLA, the brittleness increases because of increasing the degree of crystallinity (Table 1), and another aspect is the material degradation, and that is why the tensile strain at break decreases. Crystalline fractions might favour the crack propagation in the amorphous domain. In the seventh recycling cycle, a decrease in the tensile strain at the break by almost 59% was recorded for PLLA. A significant decrease in tensile strain at break for PLA was also noted by Pillin et al. [27]. In accordance with the findings of Żenkiewicz et al. [39], the results again showed the most significant changes between the moulded part made of material after first recycling cycle and the virgin material, where the tensile strain at break decreased by 28%.

The impact strength of the polymers is shown in Figure 12. It can be seen that recycled PLLA exhibit lower impact strength as compared with their corresponding virgin polymer. The impact strength of PLLA decreased by 9% in the fifth recycling cycle and by 13% in the seventh recycling cycle. The same 9% decrease in impact strength at the fifth recycling cycle for PLA was also observed by Shojaeiarani et al. [20]. However, recycled PLLA shows a higher variance in the measured values. For PHBV, the results showed that the effect of the recycling process on the impact strength was not significant, and the impact strength remained nearly constant after the recycling process. On the contrary, Shojaeiarani et al. [20] observed a decrease by 30% in impact strength in the fifth recycling cycle of PHBV. Based on these results, the assumption can be deduced that in the case of PHBV copolymer, the change in properties during its repeated recycling may be affected by the amount of valerate. The decrease in the impact strength of PLLA can be explained by a significant change in the structure of the recycled PLLA (by the lower molecular weight as indicated by the results of the *MVR* determination) due to the degradation phenomenon caused by heating and shear stress through the recycling process.

The results of the mechanical properties of recycled PHBV show the easy recyclability, which means that the product with required properties can be made of this material.

### 3.4. Effect of Recycling on Heat Resistance Testing

**Figure 13:**
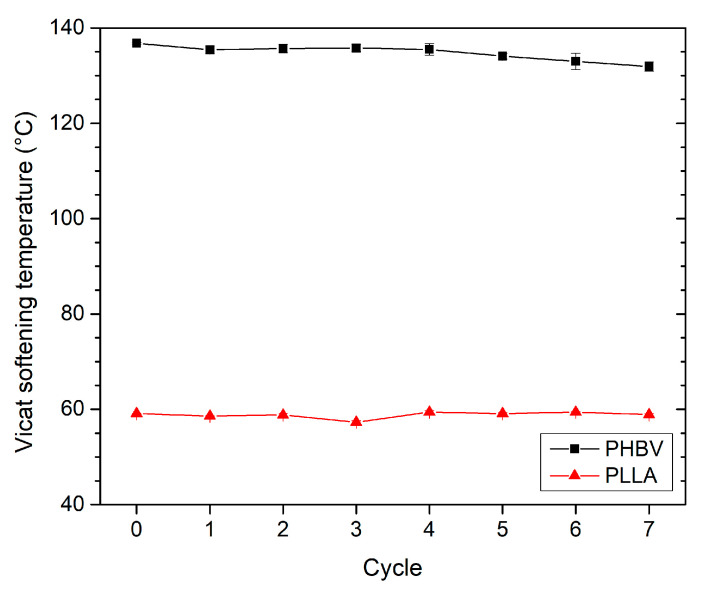
PLLA and PHBV Vicat softening temperature as a function of recycled cycle.

The heat resistance of virgin and recycled PLLA and PHBV biobased polymers was studied by determining the Vicat softening temperature (*VST*), and the results are summarised in Figure 13. For PLLA, the *VST* values remain constant during reprocessing. Considering that PLLA is a polymer with a lower degree of crystallinity, the *VST* corresponds to the glass transition temperature, which was also constant in the frame of performed analyses focused on the study of recycled PLLA. There was a slight decrease in *VST* for PHBV. In the seventh recycling cycle, the *VST* decreased by only 5 °C. This effect can be explained by the stability of the melting point and crystallinity values of the recycled PHBV (see Table 2).

## 4. Conclusions

In this work, the effect of primary mechanical recycling on the physical properties and non-isothermal crystallisation kinetics of reprocessed poly(L-lactide) (PLLA) and poly(3-hydroxybutyrate-*co*-3-hydroxyvalerate) (PHBV) biobased polymers was presented and discussed. Biobased polymers were reprocessed by milling and injection moulding in seven recycling cycles. The physical properties were investigated by differential scanning calorimetry (DSC), thermogravimetry (TGA), determination of melt volume flow rate (*MVR*), Vicat softening temperature (*VST*), and mechanical tests under static tensile and flexural loading and Charpy impact strength. The non-isothermal crystallisation kinetics during melt crystallisation and cold crystallisation were evaluated by the half-time crystallisation, and a relatively new procedure based on mathematical treatment of DSC cumulative crystallisation curves at their inflection point provides three kinetic parameters: temperature of the crystallisation start, temperature of maximum crystallisation rate and the numerical value of the maximum crystallisation rate, and final crystallinity after cooling.

This study confirmed the premise that recycled PLLA and PHBV undergo structural changes, which result in molecular modification of structure initiated by the reprocessing and repeated heating and mechanical stress of the material. This conclusion is based on the increase in the melt flow properties of the biobased polymers and the decrease in the initial degradation temperature determined by the TGA method. In the seventh recycling cycle, the flow properties increased six-fold for PLLA compared to the level reached by the virgin material in the form of pellets (before injection moulding process) and almost three-fold for PHBV. In the case of PLLA, the onset of thermal degradation of recycled material was reduced by up to 80 °C comparing the level reached by the material sampled from the moulded part moulded from the virgin material (after injection moulding), while in PHBV, there was a decrease by, at most, 10 °C. The structural changes were the most pronounced in PLLA among the studied materials. The results of DSC analysis are revealed as follows. While with reprocessing PHBV, there was only a negligible increase in melt crystallisation temperature and maximum melt crystallisation rate, and the overall degree of crystallinity remained constant; with PLLA, there was a significant change in crystallisation kinetics. As the number of recycling cycles increased, so did the start melt crystallisation temperature, the maximum crystallisation rate (by up to 2.7 times), and the melt crystallisation enthalpy (almost 10 times). Faster crystallisation from the melt reduced the risk of the polymer recrystallisation that is induced by additional heating of the moulded part. The results also provide the findings that the cold crystallisation of PLLA, which occurred after the first recycling cycle, was prevented by slow laboratory cooling. With the gradual recycling, the endothermic transformation associated with the melting of crystals with low thermal stability or with the physical ageing of the rigid amorphous fraction was also reduced. These changes are most likely caused by the polymer chain cleavage, and these chain fragments act furthermore as nucleation centres in the melt crystallisation region but do not affect the cold crystallisation, where the results showed that the maximum rate of cold crystallisation does not change with biobased polymer reprocessing. Still, the onset temperature of cold crystallisation was lowered. Significant structural changes in PLLA were also reflected in its mechanical properties. In the case of reprocessed PLLA (after seven recycling cycles), the tensile strength was reduced by ~22%, the flexural strength dropped even by 46%, the impact strength of Charpy was reduced by ~13%, and the total elongation of this naturally brittle polymer (tensile strain at break) was decreased by 59%. On the contrary, changes in the bending and tensile modulus appear to be insignificant. When measuring the glass transition temperature by DSC, the findings showed that values did not change with repeated recycling of PLLA, resulting in the fact that the Vicat softening temperature was also constant throughout the analysed recycling range of PLLA. In contrast, a slight decrease in VST by 5 °C was observed for reprocessed PHBV. The performance of this biopolymer was very consistent throughout the entire analysed range of recycling cycles with minimal changes in mechanical properties. The results suggest that PHBV processing waste, unlike PLLA, is suitable for recycling as a reusable additive to the virgin biopolymer, at least for the applications with no risk of exposure to significant degradation processes.

## Figures and Tables

**Table 1:** Thermal properties and crystallinity degree of PLLA as a function on the number of recycling cycles.

Recycling Cycle	First Heating				Cooling		Second Heating						
	Δ*H_cc_* (J/g)	Δ*H_pc_* (J/g)	Δ*H_m_* (J/g)	*X_c_* (%)	*T_p,c_* (°C)	Δ*H_c_* (J/g)	*T_p,cc_* (°C)	Δ*H_cc_* (J/g)	*T_p,pc_* (°C)	Δ*H_pc_* (J/g)	*T_p,m_* (°C)	Δ*H_m_* (J/g)	*X_c_* (%)
0	31.3	6.6	51.1	12.5	100	4.4	97.8	27.1	158	6.0	174	51.3	17.2
1	29.7	5.8	52.8	16.3	101	38.1	-	-	163	1.4	175	49.0	44.9
2	30.6	5.4	53.7	16.7	107	39.6	-	-	163	0.6	174	48.9	45.6
3	28.7	5.2	54.1	19.1	107	39.4	-	-	163	0.9	174	49.3	45.6
4	29.8	4.9	54.3	18.5	108	41.1	-	-	-	-	174	50.4	47.6
5	30.3	4.7	53.6	17.5	111	41.6	-	-	-	-	175	54.0	51.0
6	30.2	4.6	54.6	18.7	110	42.5	-	-	-	-	175	54.9	51.7
7	30.7	4.4	54.8	18.5	110	42.4	-	-	-	-	174	55.5	52.3

**Table 2:** Thermal properties and crystallinity degree of PHBV as a function on the number of recycling cycles.

Recycling Cycle	First Heating		Cooling		Second Heating		
	Δ*H_m_* (J/g)	*X_c_* (%)	*T_p,c_* (°C)	Δ*H_c_* (J/g)	*T_p,m_* (°C)	Δ*H_m_* (J/g)	*X_c_* (%)
0	97.2	60.1	122	85.7	172	97.2	66.6
1	97.7	60.0	123	86.8	170	97.7	66.9
2	96.3	60.0	123	86.7	172	96.3	66.0
3	87.2	60.0	123	86.7	171	87.2	66.6
4	97.5	60.7	123	86.8	170	97.5	66.8
5	97.0	60.9	124	86.8	171	97.0	66.4
6	97.0	60.4	124	86.8	170	97.0	66.4
7	98.2	61.2	124	87.8	171	98.2	67.2

**Table 3:** Thermogravimetric analysis of PLLA and PHBV as a function on the number of recycling cycles.

Polymer	Degradation Temperature	Recycling Cycle							
		0	1	2	3	4	5	6	7
PLLA	*T_5_* (°C)	343	332	324	272	263	327	269	272
	*T_max_* (°C)	370	368	367	349	348	366	345	346
PHBV	*T_5_* (°C)	281	281	278	279	278	271	273	275
	*T_max_* (°C)	298	298	296	297	296	292	294	293

**Table 4:** Crystallisation half-time (*t*_0.5_), crystallisation starting temperature (*T_S_*), inflection point temperature (*T_i_*), relative crystallinity at the inflection point (*X_Ti_*), and maximum crystallisation rate (*s_i_*) during melt crystallisation of PLLA and PHBV as a function on the number of recycling cycles (cooling rate: 10 °C/min).

Recycling Cycle	Melt Crystallisation of PLLA					Melt Crystallisation of PHBV				
	*t*_0.5_ (min)	*T_s_* (°C)	*T_i_* (°C)	*X_Ti_* (°C)	*s_i_* (1/°C)	*t*_0.5_ (min)	*T_s_* (°C)	*T_i_* (°C)	*X_Ti_* (°C)	*s_i_* (1/°C)
0	2.81	108.6	100.8	44.1	5.6	1.21	124.6	122.7	39.9	21.0
1	2.50	111.5	106.3	48.9	9.4	1.24	125.0	123.3	36.2	20.5
2	2.26	112.3	107.3	49.9	10.3	1.24	125.6	123.3	42.7	19.2
3	2.17	111.7	106.3	54.4	10.0	1.21	125.5	123.5	41.7	20.9
4	1.94	112.4	108.4	47.1	11.7	1.23	125.8	123.5	43.4	18.5
5	1.61	114.5	111.5	47.6	15.7	1.24	125.9	123.8	40.6	19.6
6	1.68	113.9	110.6	49.3	15.0	1.27	126.3	124.5	37.1	20.4
7	1.70	114.0	110.9	46.8	15.3	1.21	126.2	124.6	36.8	22.1

**Table 5:** Cold crystallisation temperature (*T_p,cc_*), cold crystallisation starting temperature (*T_s,cc_*), inflection point temperature during cold crystallisation (*T_i,cc_*), relative cold crystallinity at the inflection point (*X_Ti,cc_*), and maximum cold crystallisation rate (*s_i,cc_*) of PLLA as a function on the number of recycling cycles.

Recycling Cycle	Cold Crystallisation of PLLA—First Heating				
	*T_p,cc_* (min)	*T_s,cc_* (°C)	*T_i,cc_* (°C)	*X_Ti,cc_* (°C)	*s_i,cc_* (1/°C)
0	97.5	101.0	98.0	49.9	16.7
1	95.4	98.1	95.2	49.1	16.9
2	94.6	97.5	94.7	49.1	17.6
3	94.1	97.2	94.4	47.3	17.1
4	93.2	96.2	93.4	49.9	17.5
5	93.4	96.6	93.6	50.6	16.8
6	92.4	95.6	92.5	49.0	16.0
7	92.5	95.4	92.6	48.1	16.9

## Data Availability

Not applicable.
